# An Initial Passive Phase That Limits the Time to Recover and Emphasizes the Role of Proprioceptive Information

**DOI:** 10.3389/fneur.2018.00986

**Published:** 2018-11-22

**Authors:** Maeva Le Goic, Danping Wang, Catherine Vidal, Elodie Chiarovano, Jennyfer Lecompte, Sebastien Laporte, Jacques Duysens, Pierre-Paul Vidal

**Affiliations:** ^1^COGNAC-G (COGNition and ACtion Group), Université Paris Descartes–CNRS UMR-MD–SSA, Paris, France; ^2^Institute of Information and Control, Hangzhou Dianzi University, Hangzhou, China; ^3^Plateforme d'Etude de la Sensorimotricité, Université Paris Descartes, Paris, France; ^4^Arts et Metiers ParisTech, Institut de Biomecanique Humaine Georges Charpak, Paris, France; ^5^Movement Control and Neuroplasticity Research Group, Department of Kinesiology, KU Leuven, Leuven, Belgium

**Keywords:** accidental fall, disequilibrium, stability, postural control, perturbation, sensory information, biomechanics, multi-joint kinematical model

## Abstract

In the present experiments, multiple balance perturbations were provided by unpredictable support-surface translations in various directions and velocities. The aim of this study was to distinguish the passive and the active phases during the pre-impact period of a fall. It was hypothesized that it should be feasible if one uses a specific quantitative kinematic analysis to evaluate the dispersion of the body segments trajectories across trials. Moreover, a multi-joint kinematical model was created for each subject, based on a new 3-D minimally invasive stereoradiographic X-ray images to assess subject-specific geometry and inertial parameters. The simulations allowed discriminating between the contributions of the passive (inertia-induced properties) and the active (neuromuscular response) components during falls. Our data show that there is limited time to adjust the way one fall from a standing position. We showed that the pre-impact period is truncated of 200 ms. During the initial part of a fall, the observed trajectory results from the interaction between the destabilizing external force and the body: inertial properties intrinsic to joints, ligaments and musculotendinous system have then a major contribution, as suggested for the regulation of static upright stance. This passive phase is later followed by an active phase, which consists of a corrective response to the postural perturbation. We believe that during a fall from standing height, it takes about 300 ms for postural responses to start correcting the body trajectory, while the impact is expected to occur around 700 ms. It has been argued that this time is sufficient to change the way one falls and that this makes it possible to apply safer ways of falling, for example by using martial arts fall techniques. Also, our results imply visual and vestibular information are not congruent with the beginning of the on-going fall. This consequence is to be noted as subjects prepare to the impact on the basis of sensory information, which would be uniquely mainly of proprioceptive origin at the fall onset. One limitation of the present analysis is that no EMG was included so far but these data are the subject of a future study.

## Introduction

Falls are a threat to the health and independence of the older part of the population. In this context, it is crucial to know how far it is possible to adjust the way a person falls, in order to prevent damage, or in the best case, in order to guarantee recovery. This is even more so in patients. For instance, the reduced ability to accurately adjust foot placement during walking in individuals with focal cerebellar lesions appears to be a movement control deficit, which could contribute to increased fall risk (1).

Upright human stance is considered as an unstable multi-articulated system which has to face a constant but disturbing force acting on the body: gravity. In the absence of stabilizing torques controlled by the postural system involving both active and passive mechanisms to maintain upright body stance, a fall would occur (2, 3). The phase of the fall preceding the ground impact is crucial for preparation of landing, but it lasts no more than 750 ms in a standing subject (4, 5).

It is argued here that it is possible to subdivide this period into two phases, depending on the absence or presence of body corrective movements. In the first period, the movements of the various body parts are very stereotyped and mostly explained by inertia. This part is termed therefore “passive,” defined as devoid of evidence of active involvement of corrective movements. It is hypothesized that during this part, there is very little dispersion of displacements of the head when individual reactions to the same perturbation are compared. This inertial phase may ultimately determine the ability to trigger efficient muscle activities as it potentially leaves a short time-window available to actively compensate a loss of balance. However, the mechanical behavior of the body in reaction to an external destabilization has not generated much interest even though it may be important for the availability and redundancy of the sensory information upon which the subjects prepare to the impact (6). In contrast, in the second part of the fall, the displacements are expected to show large variability as corrective motor strategies are displayed. This part will be termed “active.” This subdivision is valid as long as the reactions are mostly due to feedback. This requires a protocol in which randomization of conditions prevent feedforward mechanisms, as is the case in ecological conditions when perturbations are unexpected. The value of this approach is that one can obtain insight in the time scale required for appropriate corrective movements (7).

Some authors claimed that the passive mechanisms arising from biomechanical properties of the musculo-articular system (muscle tissues, aponeurosis, synovial fluid, ligaments, articular capsule, joint friction, skin) or the muscle-tendon unit (visco-elasticity, damping, stiffness) help to counter gravity forces and maintain balance (8) without necessarily a continuous muscle activity (9) in the same way a spring resists when displaced from its resting equilibrium (10). This mechanical view of postural regulation was opposed to a neurological model (2, 11) which favors a postural regulation via an internal model based of sensory inputs that detect CoP movements and result in the control of CoM displacement. The question whether both contributions of passive properties and active control mechanisms to maintain upright stance while a perturbation occurs (floor tilts, moving scenes, or galvanic stimulation) are independent from each other remains uncertain (12). Some studies also suggest the existence of an ≪ effective time delay ≫ based on an independent channel model that may even not be linked to delays in neural processing, transmission of information nor muscle activation time (11)

To explore these issues, one needs experiments that include high-threatening perturbations i.e., challenging enough to induce real and non-recoverable falls. Such experiments can be performed by suddenly shifting a platform that is sufficiently wide to allow falls or corrective responses. In a ground-breaking study, Hsiao and Robinovitch (4) disturbed the balance of young adults standing on large mattresses that were translated quickly. They found that it takes approximately 700 ms before touchdown. Review of their stick-figure animations revealed that active movements appeared to start some 300 ms after fall onset but this aspect was not studied in detail. Similarly, except for hip and wrist, the body segment movements were only documented with stick diagrams without detailed analysis. The diagrams in this article seemed to suggest that the head did not move in the first 150 ms of the fall but more specific information was not available.

Such information on head motion is important if one wants to judge the contribution of vestibular reflexes in balance corrective responses. The role of the vestibulo-spinal reflex is relatively clear in responses to free fall (13), but it remains largely unknown for surface-translation type studies. In forward falls after tether release the head starts to move within 10–20 ms, hence one could expect a contribution of the vestibular system to balance-correcting responses in lower leg muscles, occurring as early as 60 ms after the stimulus (14). Yet the onset of the responses was identical in patients with vestibular loss. Similarly for experiments with a moving support surface, Allum and Honegger (15) showed that vestibular loss caused no change in the amplitude of balance-correcting responses. These and similar data (16), question the role of vestibular inputs in fall-recovery but the definite proof requires a detailed examination of the head movement during platform translations. Another reason to study head motion in experiments with a moving platform is that it allows to judge the functional contribution of early muscle activations, provided by stretch reflexes, automatic postural responses, and startle. If these responses influence the fall behavior in providing stiffness and generate an appropriate torque at ankle joint, then it is also important to see from what point in time it affects the head trajectory.

During a fast support translation triggering a potential fall, it is proposed that these responses have little effect in the “passive” phase. Rather it is probably the delayed component (long latency responses) of muscle activations occurring in the “active” phase which help to compensate substantial balance disturbances and determine the outcome of the fall or recovery (17). This is reminiscent of the responses to tripping where it was found that early EMG activations (up to 100 ms) did not correlate with the behavioral response [elevating or lowering strategy, (18)].

In the present experiments on a large sample of subjects, multiple balance perturbations were provided by unpredictable support-surface translations in various directions and velocities. The aim of this study was to distinguish the passive and the active phases during the pre-impact period of a fall. It was hypothesized that it should be feasible to evaluate the dispersion of the body segments trajectories across trials. Moreover, a multi-joint kinematical model was created for each subject, based on a new 3-D minimally invasive stereoradiographic X-ray image. The latter were used to assess subject-specific geometry and inertial parameters. The simulations allowed disentangling the contributions of the passive and the active components during falls. Finally, the present study is relevant in the context of perturbation training. It was proposed that such training could be valuable to facilitate generalization of effective responses to various perturbations (19). If so, the relevant question arises as to what number of repetitions of multidirectional perturbations is needed to obtain such beneficial generalization.

A follow-up study (unpublished data) focus on which strategies are used to successfully avoid falling.

## Materials and methods

### Participants

The ability to react to sudden perturbation was investigated in 23 healthy, young and physically active volunteers (9 women and 14 men, 28.6 ± 8.2 years). All participants were free of any diagnosed diseases that may have affected their control of balance or limb movement. Subjects were normal bodied (172.3 ± 8.2 cm and 66.1 ± 8.8 kg) and selected in order to cover a representative range of anthropometric properties. All but two were right-side dominant. Their body mass index (22.3 ± 2 kg/m2) corresponded to a “normal” range “body mass index (BMI) classification” and “Global Database on Body Mass Index” (20). The participants' levels of physical activity was assessed by asking them whether they practiced more or < 3 h of endurance exercise per week (21). All experiments were performed according to the Declaration of Helsinki, and the experimental procedures were approved by the Human Ethics Committee on Human Research of the University of Pierre-et Marie-Curie (CPP 06036). All the subjects provided written informed consent prior to their participation.

### Apparatus and procedure

While subjects were standing upright quietly in a standard position with their eyes open, balance was disturbed using a servomotor controlled movable platform driven by a pneumatic piston. The perturbation was provided by sudden multidirectional horizontal translation of the support surface in one block of 32 trials. The amplitude of displacement was 40 cm and the imposed waveform was a ramp. These translations were randomly presented either sideways (rightward, leftward) or in the anteroposterior (forward, backward) directions. Postural control was further challenged as two magnitudes of perturbation were randomly applied in combination with each direction: a low-threatening perturbation (mean velocity: 35 cm/s, peak acceleration value: 7.8 m/s2) and a high-threatening perturbation (90 cm/s; 10.78 m/s2). These two velocity ranges were selected on the basis of pilot trials to ensure successful recovery in about 80% of the time in “slow” trials whereas “fast” perturbations were sufficiently challenging to trigger non-recoverable falls. Some unpredictable aspects of a fall were a prerequisite to design our protocol, such that no training trials were given and the trials were randomized. The body movements were quantified from the first impulse, and the instant at which the perturbation was delivered as well as its velocity and direction were unknown to the subjects. No specific instructions were given with regard to the postural reaction. A standard initial position (12 cm spacing between heels, 10 deg angle between the medial margins) was used in all trials. At all times, participants were securely harnessed in order to abort a complete fall, without otherwise restricting movement in the first 500 ms. The inter-trial time interval was dictated by participant readiness and platform resetting time.

### Data collection

The onsets of platform translation as well as displacement of the body segments were calculated as the first inflection above 2 standard deviations (SD) from the baseline displacement for each individual trial. All timing measures were defined relative to this perturbation onset (PO).

#### Kinematics

Body kinematic data were collected at a sampling rate of 200 Hz using a three-dimensional motion-capture system (Codamotion-CX1 system, Charnwood Dynamics, and Leicestershire, UK) with a spatial resolution of 0.3 mm. Four Coda CX1 unities tracked the coordinates of 27 infrared active LED markers placed bilaterally on the anatomical landmarks: head of the fifth metatarsal (“toe”), head of the first metatarsal, lateral malleolus (“ankle”), external and lateral femoral condyles (“knee”), greater trochanter (“hip”), anterior superior iliac spine (“pelvis”), zyphoid process at the lower part of the sternum and L5/S1 joint (“thorax” and “trunk”), C4 and C7 spinous processes (“neck”), left and right tragus and nasion (“head”), acromion process (“shoulder”), olecranon (“elbow”) and processus styloideus (“wrist”). One marker was placed on the platform and an accelerometer, sampling at 1,000 Hz, was fixed on the platform to calibrate the starting moment. The measured marker coordinates data, together with Dempster's anthropometric data (21) adapted by Winter (22), made it possible to determine the weighted summation of individual segments from which the trajectory of the whole Center of Mass (CoM) was derived. Marker displacement data were low-pass filtered at marker-specific optimal cut-off frequencies (range: 4.5–9 Hz) using a recursive second-order Butterworth Filter.

#### Determination of the passive phase of a fall

**Figure 1:**
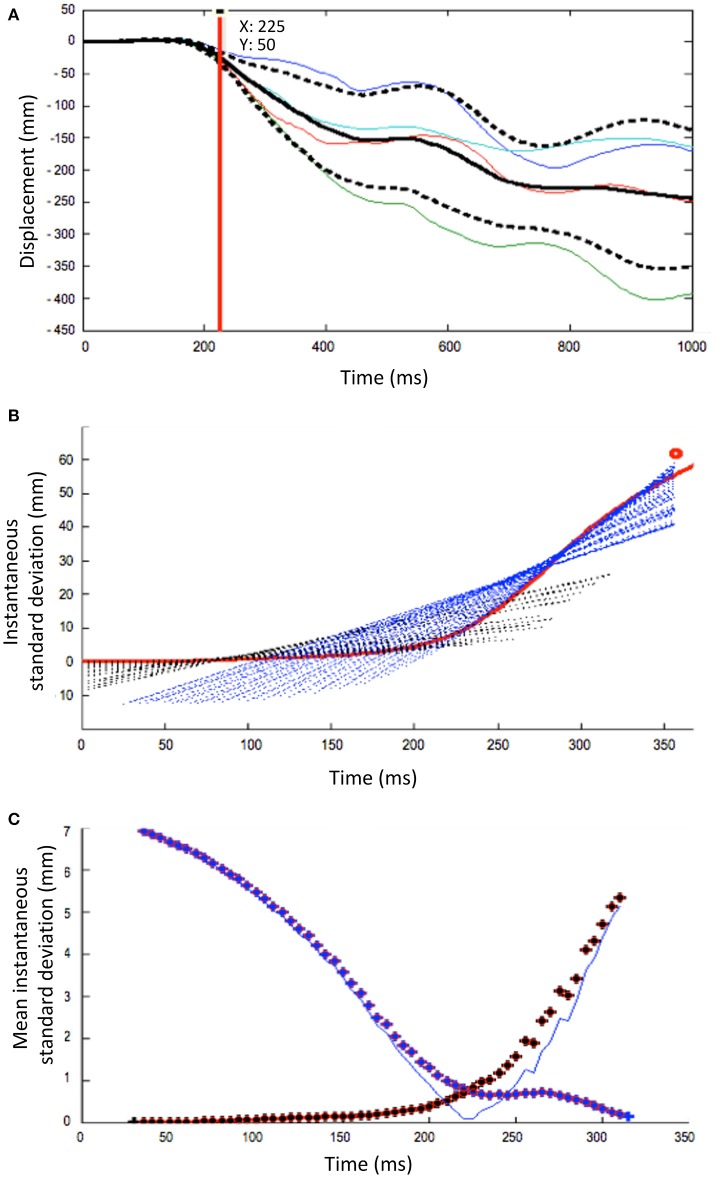
Methodological process to determine the increase of the inter-trial variability at a given condition for a single subject. The illustration is here based on the head segment displacement after a forward fast platform translation. **(A)** The mean displacement (black solid line) ± 1 s.d. (black dotted lines) is calculated from the superposition of 4 trials (colored solid lines). **(B)** Instantaneous standard deviation curve against time (mean s.d), as used to determine inflection point (here at about 200 ms). Added are the sliding least-squares lines originating from both sides of the inflection. **(C)** Determination of the inflection point of the mean instantaneous standard deviation curve (blue squares), from which the inter-trial variability increases significantly (red squares). It corresponds to the intersection point of these lines, which is the minimal value (blue thin line).

To determine the duration of the passive phase, the onset of displacement of each body segment was first assessed. Then, for each subject and body segment, the four individual trials in a given condition were superposed (see Figure 1A). The instant at which the trajectories were considered to vary from one trial to another was determined based on the inflection point, which was based on the instantaneous standard deviation curve (Figure 1B). This point (± 200 ms) was assessed using sliding least-squares lines originating from both sides of the inflection. The intersection point of these lines was associated to a minimal value, which corresponds to the inflection point (Figure 1C).

#### Stability assessment

The positions of the markers on the toes, heels, and lateral malleolus were used to define the fore-aft and medial-lateral support boundaries. Additional length measurements were made to assess foot anthropometry, such as foot length, to evaluate the distance between the markers and the anteroposterior foot extremities (line joining the heels to the big toes). The motion state of the CoM (23, 24) in relation to the leading edge of the base of support (BoS; either the rear of the heel-for a forward translation-, the front or the lateral side) was calculated as the margin of stability, taking into account both the projected CoM position expressed relatively to the boundaries of the BoS and normalized to foot length, and the CoM velocity expressed relatively to BoS velocity. An instantaneous Time To Contact (TTC) value was calculated dividing the instantaneous distance of the CoM to the stability boundary toward which it was moving by its closing directional velocity, a first derivative of CoM positional data (25, 26). Furthermore, the trials in which the CoM motion state never reached the boundary, those in which it almost crossed it (approaching distance < 5 mm; TTClim) and finally those in which it exceeded the boundary had to be considered in order to determine quantitative measures of a person's stability. In the latter case, it characterized a backward, forward or lateral loss of balance (depending on the direction of the perturbation), with regards to the computed limits of stability (27–29). Of particular interest was the comparison between the instant at which a recovery step was initiated and this time-data point, henceforth referred to as the stability boundary.

#### Fall-recovery outcome

A putative fall was detected using three redundant criteria. Firstly, a “fall” was registered each time the subject ended in a seated position in the harness. Secondly, a fall occurred when the midpoint connecting the hip joint centers descended within 5% body height of its initial standing height (30); otherwise, the trial was classified as a recovery. Thirdly, the automated classification was checked using a video recording of the trial. The outcome of each trial was scored 0 for recovery or 1 for a fall; all trial scores were added up to calculate a subject's “Fall score.”

### Postural strategies

Different strategies have been identified in the literature, among which we can mention the feet-in-place strategy, such as the hip and ankle strategies, and the change of support strategy such as stepping. As explained above, the trials were first distinguished as successful recovery or fall and further qualified by the strategy in use and its efficiency. The methodological approach is presented here although the active phase analysis is the topic of future study (unpublished data). Trials including at least one step were identified through different sources: the observation of the presence of steps during the experimentation supported by the control video, the movement of the feet detected by the motion capture analysis (both a toes and ankle marker displacement recorded along the vertical axis). Further analysis consisted in identifying a single vs. multiple steps strategy and the use of a so-called “cross over” vs. a “side step” (loaded or not) in mediolateral trials (31). Several parameters were then analyzed to better characterize the steps: the instant of step initiation or “foot off” (determined as the first sample after PO in which the ankle marker started moving in upward direction), the step duration and length (evaluated with the determination of the instant of touch down), the height of the step (maximum value reached on the z axis), the preferential limb used for the first step and the stability margin at step initiation and the number of steps. In addition, among other measures used to quantify modifications in the subject's response strategies to the perturbation, angular kinematics were calculated through onset times, peak amplitude and peak velocity of the following joint motions: ankle {flexion, extension, pro-supination}, knee {flexion, extension}, hip {flexion, extension, abduction}, trunk {forward and lateral flexion/extension}, shoulder abduction, elbow flexion, neck {forward and lateral flexion, rotation} and finally, the head linear and angular displacement in space. These angular displacements were evaluated according to the initial state calculated over the 2,000 ms preceding PO: a comfortable vertical upright position, arms at sides, with forearms naturally rotated in a relaxed posture {pronation}.

### Fall modeling

An accurate 3D personalized model of each subject was built from biplanar (anteroposterior-AP and lateral-LAT) stereoradiographic images of their whole body using the low dose technological X-ray system EOS® (Biospace Instrument, Paris, France). Specific 3D reconstruction methods—based first on an identification of specified 2D anatomical marks and contours digitized in both radiographs, then on a fast computation of a generic model followed by local deformations—made it possible to assess accurately subject-specific geometry and each body segment inertial parameters (32–34). The body shape reconstruction was divided into 11 segments: head, neck, thorax, abdomen, hip, thighs, legs, and feet. The segment boundaries were those described by Dumas (35), and by Sandoz (36) for the neck and the abdomen. For each body segment, the masses, 3D CoM location and inertial matrixes were calculated thanks to specific software developed using Matlab and densities as derived from the literature (21, 37). Because of inside air, lung density was defined in order to have a global density of the thorax (lungs and all the other organs) in accordance with the literature. As the 3D reconstruction was not yet completed for the upper limbs, they were represented by rigid bodies and reconstructed using DLT algorithms based on the digitization of anatomical landmarks such as acromion, olecranon, wrist joints, and fingertips. The masses and CoM location of the arm, forearm and hand were assessed according to the Dempster database. The total body mass was calculated by the addition of the masses of each virtual body segment. The global body CoM was defined as the weighted barycenter of all segmental CoM. This whole-body reconstruction method was established for a standing subject (36).

**Figure 2:**
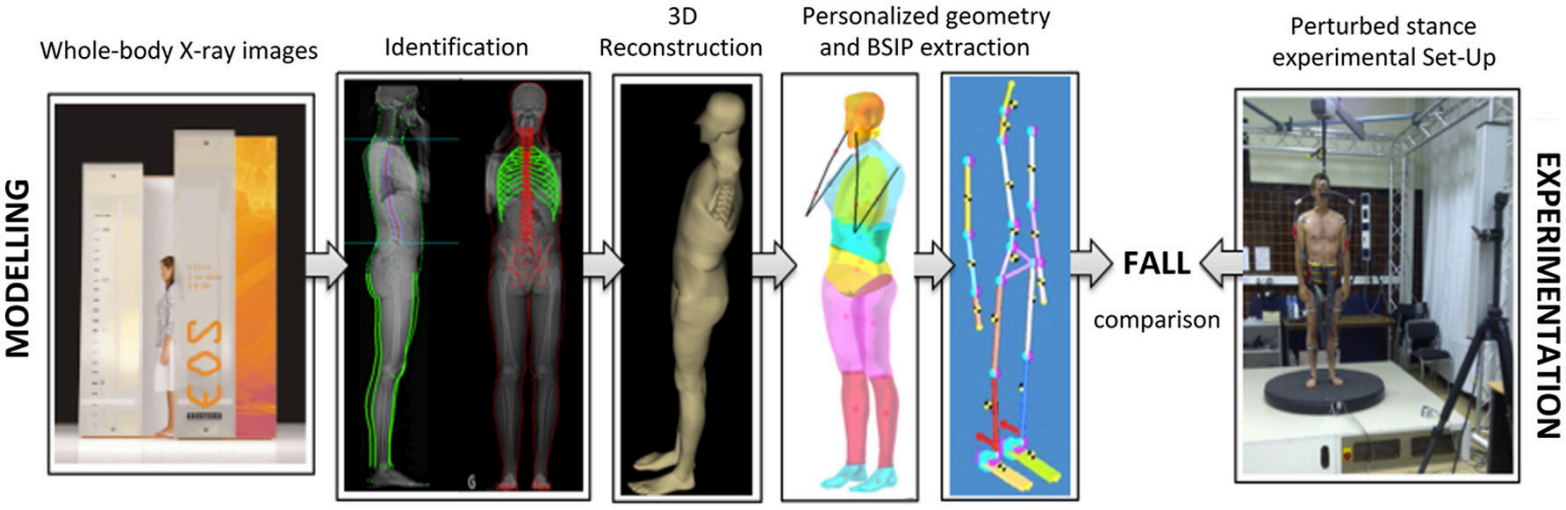
Kinematic comparison of a real *vs*. a theoretical fall in human subjects (experimental data) and their personalized mechanical models (simulation data), respectively.

In a second step, these parameters served as inputs for the following numerical and personalized model including 17 rigid segments (head, neck, thorax, abdomen, pelvis, arms, forearms, hands, thighs, legs, and feet) connected with 16 revolute or ball joints offering 92° of freedom. Its simulated movement after the same imposed destabilization as used in the experimental part (in terms of perturbation nature, direction, velocity, and acceleration) served as a database for a comparison with the kinematic behavioral data collected during the experiments on the movable platform. Outputs included the displacements of each segment's center of gravity (x, y, z components) in the global coordinate system, the translations and rotations of the head markers and local frame of reference and the angular displacements described earlier. Figure 2 summarizes the whole procedure.

### Statistical analyses

Statistical analyses were performed using the Statistical Analysis Software (SAS) for Windows. All means throughout this paper are given with their associated standard deviation (mean ± SD). The groups were compared using the chi-squared or Fisher test for categorical data (age, weight, height, direction, velocity). A general linear model repeated-measures ANOVA with the Bonferroni correction for multiple comparisons was used to compare the differences between falls and recoveries for each recorded dependent variable (38).

The comparison of individual characteristics between the two groups (Fall vs. Recovery) was analyzed by a Wilcoxon Rank Scores test for continuous variable (age, weight, height, and BMI); and by a Fisher test for categorical variables (gender and fitness level). Then, in a base including all the tests combined, a Chi2 test (1 ddl law) was used to study the effect of direction and speed on fall occurrence. A general linear model with repeated measures (subjects) was used to compare calculated variables such as latency of movement, steps characteristics, spatiotemporal aspect of muscle activity. Indeed, a classical analysis was not possible in our main experimental study: the samples were not independent and did not contain an identical number of falls and recoveries by subject (paired study). Intra-individual and inter-individual variability were evaluated using a mixed model. Adjustments on the subject, the number of steps, and the experimental condition were made. We thus conducted an analysis in subgroups i.e., separate analyzes by direction and speed (experimental condition). Further planned contrasts isolated effects with the Bonferroni correction were then performed to compare two by two the differences between conditions.

For kinematic analyzes, we used a non-parametric paired-averaging test, the Wilcoxon test.

The level of statistical significance for all tests was *p* < 0.05.

## Results

From the 25 subjects, a total of 526 trials were investigated in detail and considered for the main analysis. These trials were unambiguously classified as a successful or a failed recovery (i.e., a fall). The other 210 trials were excluded from statistical analyses due to excessive movement occurring prior to the perturbation, missing marker data, harness rope-assisted behaviors or platform translation abnormalities.

### Occurrence of falls and recoveries

**Figure 3:**
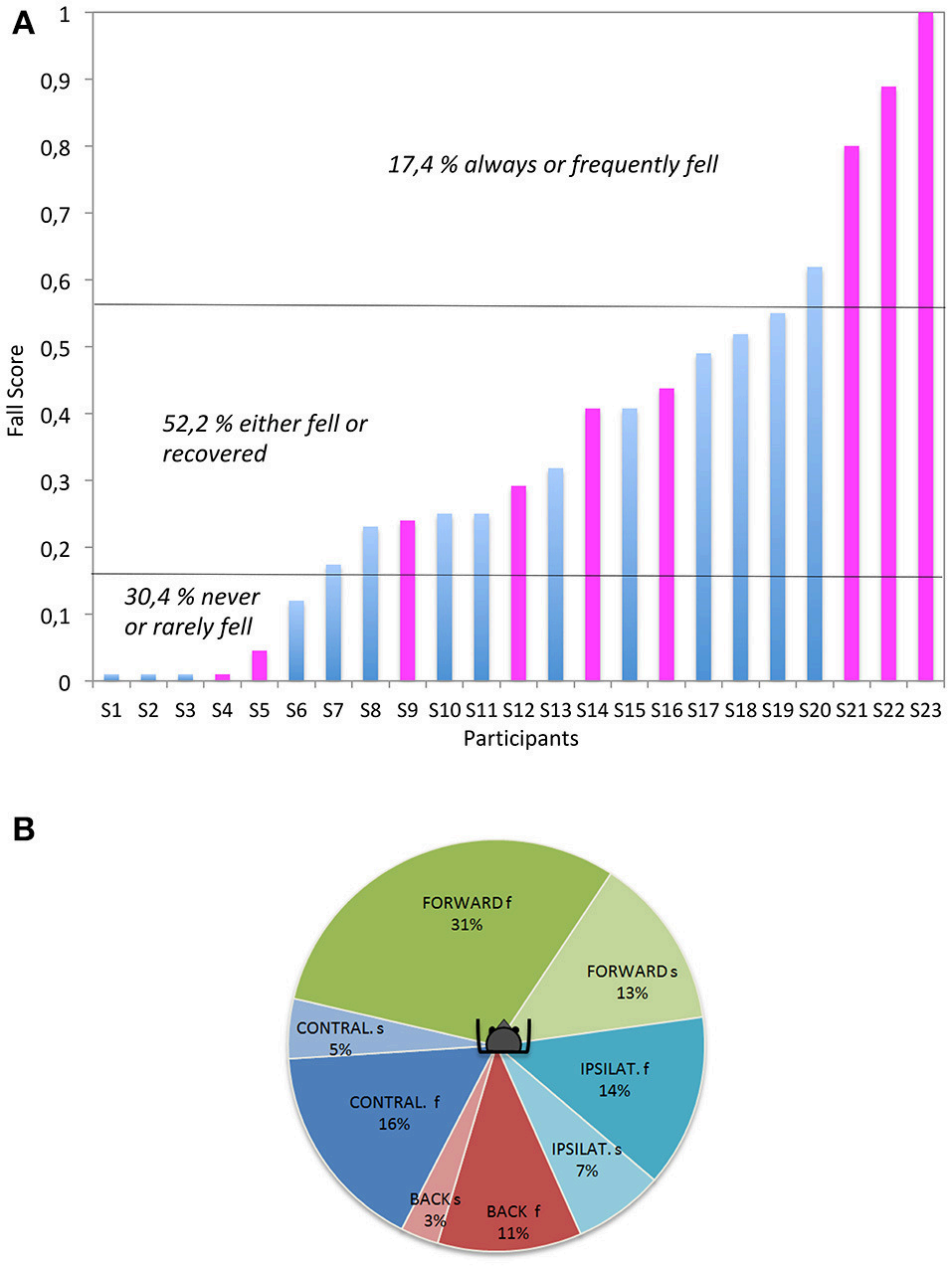
(**A**, left): Variability of the subject (Si) performances. The individual Fall Scores are averaged for each subject and all experimental conditions (blue and pink colors: male and female, respectively). (**B**, right): Fall occurrence according to the perturbation direction and velocity. f, fast; s, slow, IPSILAT. and CONTRAL. refer to the mediolateral directions, relative to the dominant leg (hence ipsilateral refers to a platform translation toward the dominant leg).

As mentioned in the Materials and Methods section, a fall score was calculated for each subject separately in order to describe inter-individual variability in the trial's outcome (i.e., fall vs. recovery). A fall score (FS) of 1 indicated that the subject had fallen at every trial, whether it was a slow or a fast translation. On the other hand, a low FS was associated to few falls across all the subject's trials. Figure 3A illustrates the large between-subject variability as the subjects could arbitrary be divided in three groups: those who never or rarely fell (30.4% with a FS < 0.15), the ones who always or frequently fell (17.4% with a FS > 0.5), and the remaining majority of individuals (52.2%) who had variable FS as they either failed or successfully managed to recover their balance. Within this group, the outcome variability (Fall (F) vs. Recovery (NF) amongst subjects was not related to age (*p* = 0.85, Wilcoxon test), gender (*p* = 0.2, Fisher test), height (F: 169.5 cm vs. NF: 173.2; *p* = 0.23, Wilcoxon test), weight (F: 63.6 kg vs. NF: 67.2; p = 0.85, Wilcoxon test), BMI (F: 22.05 vs. NF: 22.62; *p* = 0.62, Wilcoxon test), or fitness level (*p* = 0.96, Fisher test).

The occurrence of a fall varied according to the direction of platform movement (*p* = 0.0003, Chi2). As summarized in Figure 3B, forward trials (44% of falls) appeared to be significantly more challenging than backward (14%) and mediolateral trials (21%). As a rule, fast trials generated significantly more falls than slow translations (*p* < 0.0001, Chi2), with the exception of ipsilateral trials i.e., when the perturbation occurred toward the side of the dominant leg (*p* = 0.06, Chi2).

Hence, speed was clearly important. Out of 266 fast trials, 47% led to a fall with 31% occurring for the fast forward platform translation. The second most challenging condition was fast contralateral [i.e., when the perturbation occurred contralateral to the side of the dominant leg (*p* = 0.06)], leading to 16% of falls. The failed recoveries were episodic after a backward fast translation. In contrast, much less falls were induced by the slow platform movement. Out of 260 slow trials, 18% led to a fall. The backward falls (following a forward platform translation) remained the most frequent (13%) while the second most challenging condition was the contralateral translation, leading to 6% of falls. Slow backward trials only generated 3% of falls.

**Figure 4:**
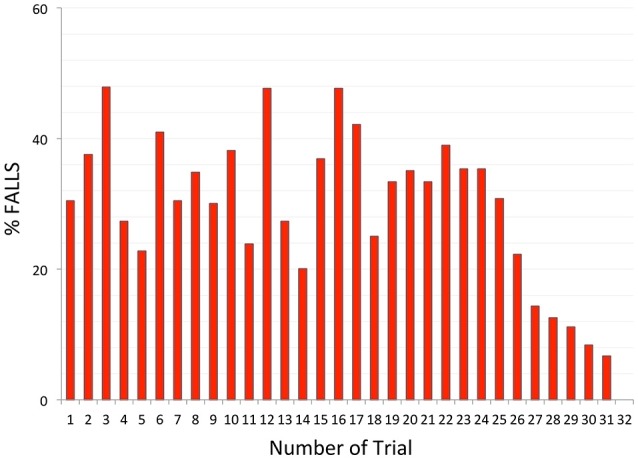
Fall occurrence according to the number of trials.

With respect to the question whether subjects are able to learn to recover balance, the data on the percentage of falls was plotted with respect to trial number. As shown in Figure 4, the occurrence of a fall did not depend on the rank of the trials during the first 25 trials. Later trials however, showed a decrease in fall rate, indicative that learning is possible but requires time and experience.

The relative absence of learning during the first 25 trials was probably related to the randomization and the unpredictability of the direction and/or speed of the upcoming disturbance. Nevertheless, the series always started with a “forward slow” trial (to limit startle reactions) and this significantly resulted in more falls than the rest of the tests in slow condition (“first trial effect”). The particular analysis of the first trial (for instance compared to a second absolute test or a second similar trial in speed and direction) for each individual constitutes an axis of improvement for future studies given its emphasis in the literature.

### The three phases of a fall

**Figure 5:**
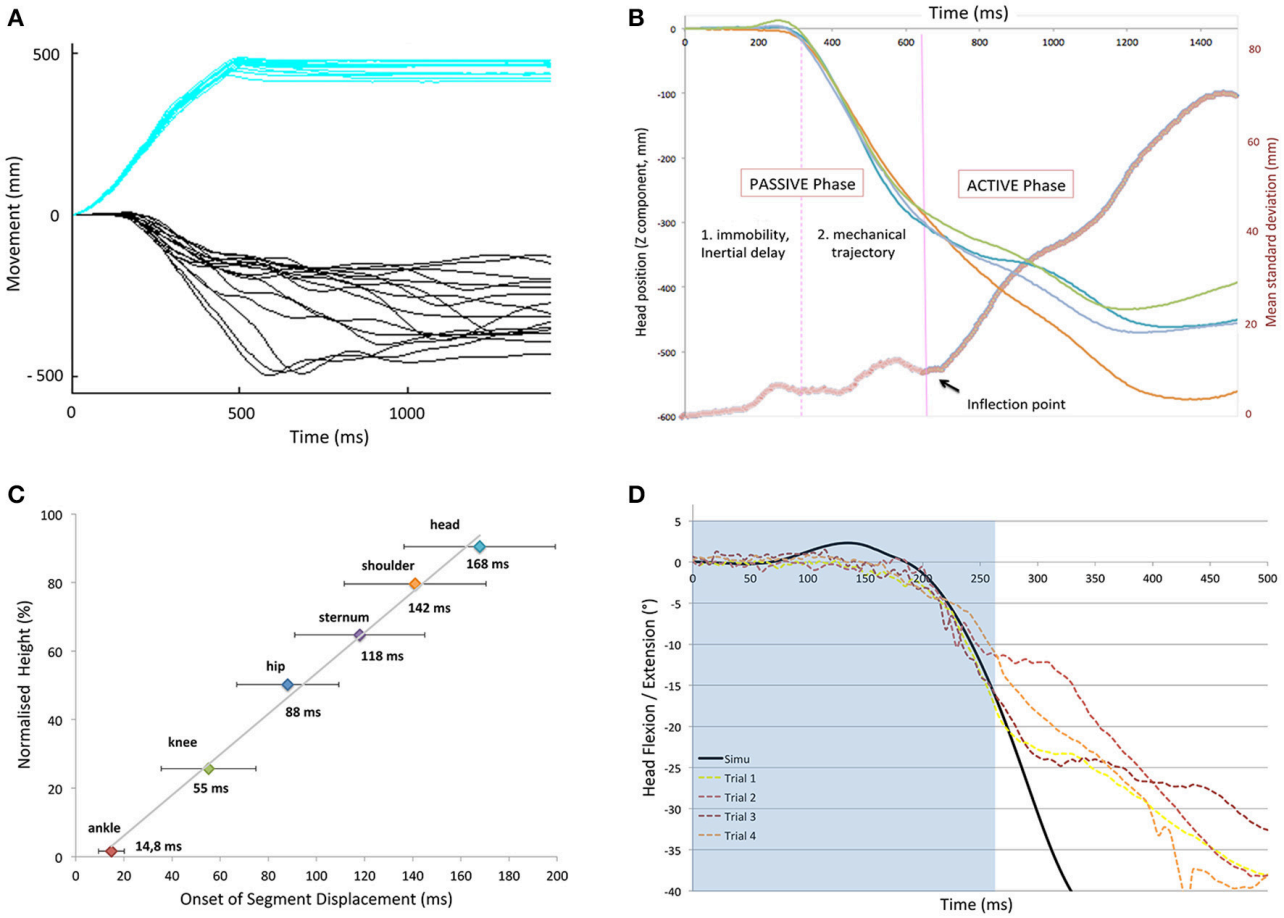
**(A)** Platform (blue) and vertical head displacements (black) during the first second of a backward fall (forward translation) for several subjects. **(B)** Illustration of representative head kinematics: superposition of four trials after a forward fast translation of the support surface and instantaneous dispersion curve (red thick line). The “variability threshold“ (separating passive and active phase), was determined using the inflection point on the curves separating the trajectories with low and high dispersion (purple line). **(C)** Onset of deviation of the various body parts with respect to height. Note that there is a linear relation between segment height and its inertial latency of displacement. **(D)** Simulated (black) vs. Experimental (colored dotted lines) data for the head displacement (z axis) of a subject after a forward fast translation. Note that both curves superimpose in the passive part (light blue background).

As pointed out in the introduction, the pre-impact phase of a fall lasts no more than 750 ms in a standing subject, so it is important to identify the timing of active corrections to counteract a loss of balance. To evaluate this timing, we used a method based on the dispersion of the traces after perturbation onset. Figure 5 illustrates this for the head segment.

Figure 5A shows that constant forward translations of the platform led to a series of head trajectories that started to diverge after some 300 ms. This divergence was further evaluated with an instantaneous dispersion curve as illustrated in Figure 5B. It shows the superposition of four trials after a forward fast translation of the support surface applied to a subject. From such displacements, an inflection point was determined on the mean instantaneous standard deviation curve and referred to as a “variability threshold,” separating the trajectories with low and high dispersion. The period after the inflection point was termed the “active phase” (T3) as it was assumed that the sudden increase in dispersion was due to subjects actively reacting to the perturbation with the whole body, including the head.

The period prior to the inflection point was termed the “passive phase” and could further be subdivided. Immediately after perturbation onset, a first phase of the fall was defined as the “immobility period” (T1) as there simply was an absence of movement, due to inertia. For the head, this immobility period could last until 200 ms, which is 1/3 of the available time to recover.

In the second phase of the fall, termed “free-fall period” (T2), the head did move but very consistently on a trial-to-trial basis, suggestive of passive motion. This reproducible low-variability was observed at all levels: once each segment started to move, they followed a similar trajectory on a trial-by-trial basis (reproducible low-variability). Each trial could be divided into these three phases, whether it ended in successful recovery or not.

Each body segment followed this chronological subdivision, but with different time-intervals. Figure 5C illustrates the onset of displacement of each body segment under study, in relation to its respective height, after a forward fast support surface translation. It shows the linear relation between the segment distance to the point of perturbation application. There was a clear toe-to-head progression despite an increasing variability according to height. This variability may be explained by the specific inertia of each body segment, as belonging to a group of heterogeneous solids. This linear relation was observed in each condition of perturbation (whatever the direction and velocity; r = 0.95, SD = 0.027).

The upper trunk and the head had the longest latencies. In particular, after a fast forward perturbation (backward fall), they remained motionless for T1 = 142 ms (SD 20) and 168 ms (SD 24), respectively. These T1 latencies were significantly longer than after a slow forward translation (T1 = 78 ms (SD 12) and 148 ms (SD 24), *p* = 0.018 respectively). Taken together over all velocities and directions, T1 lasted 106 (SD 22) and 142 ms (SD 31) for the trunk and head, respectively. The duration of T2 was similar across all fast and slow trials whatever the direction and lasted 92 ms (SD 33) and 105 ms (SD 38), respectively for the trunk and the head, with the exception of mediolateral trials (trunk T2 phase shortened to 75 ms; SD 26). Conversely, the head was displaced earlier after a fast mediolateral translation (*p* = 0.03) compared to slow medial-lateral trials.

T3 duration was not calculated, as it was either truncated by landing in the harness (in falls), or characterized by a return to initial and stable position. No instructions were given to the subjects. However, a prevalent strategy was to respond to the imposed destabilization with a stepping reaction, occurring in 92% of the trials (unpublished study).

A global description of the body motion relied on the CoM movement analysis, in relation to the base of support displacement. The TTC (Time To Contact, see Methods) value is a calculated variable combining CoM projection and BoS positions and velocities (see Methods), which indicate the state of balance at each instant. In slow trials, the projection of the CoM stayed inside the base of support in 75% of the trials (90% in mediolateral trials). However, after fast perturbation onset, all subjects reached a state of disequilibrium around 218 ms (mean TTC, all directions taken together). The mean TTC values amounted, respectively to 235, 236, and 188 ms for forward, backward, and mediolateral fast perturbations, and were not significantly different between falls and recoveries.

### Modeling the head and trunk trajectories

As detailed in the Methods section, a 17-rigid-segment model (head, thorax, arms, forearms, hands, pelvis, thighs, legs, and feet) was personalized to fit each subject's characteristics using data recorded from a recent non-invasive tridimensional radiographic method. This model was used to simulate the postural response of the subjects following translational displacements of the basis of support identical to the experimental perturbations.

The trajectories of the head and the trunk of this purely passive model were compared to the experimental results in order to determine at what latencies these two sets of curve diverged.

The results of a representative subject are illustrated in Figure 5D for a fast forward translation. It shows that the head displacement matches the mechanical model during the first ~250 ms. The head and trunk trajectories of the model fitted nicely until the end of the passive phase defined above (T1, T2), i.e., until these trajectories became extremely variable on a trial-to-trial basis, in particular in the forward and backward directions. The similarity between the model and the experimental kinematics was less pronounced in the mediolateral direction.

### Analysis of the active phase: angular kinematics

Falls were most prominent for anteroposterior translations. After a fast-forward translation, the head, trunk and limbs first extended passively. Could there be a difference in this “passive phase” that is predictive of falls? To examine this question, we compared the body kinematics of the trials resulting in a fall with those resulting in a recovery.

**Figure 6:**
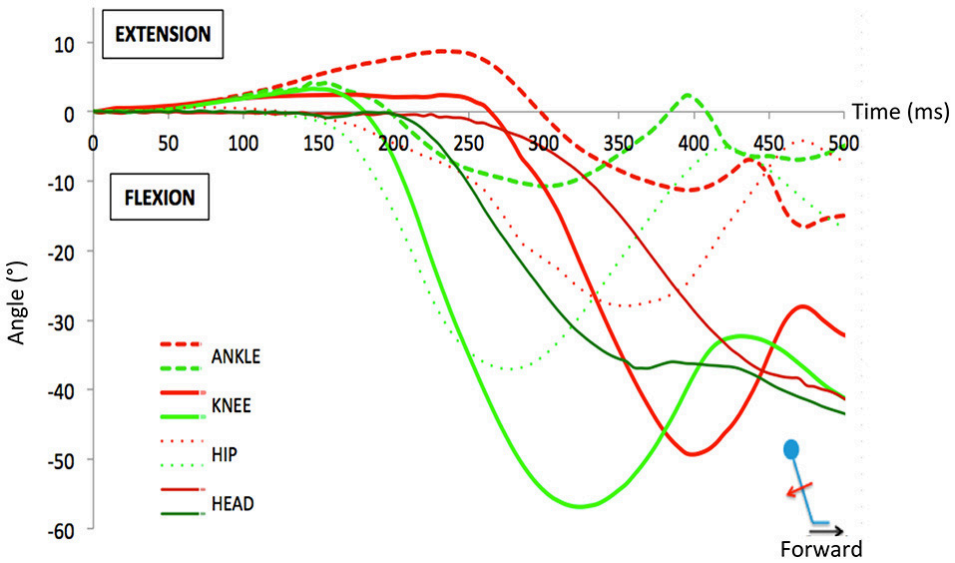
Example of the angular variations during a typical successful trial (i.e., a recovery, in green) compared to a fall trial (in red) after a fast forward translation (backward fall). During the passive phase, one observes that the limbs extend at the knee and ankle joint levels similarly in both trials for the first 160 ms. However, the extension lasted longer in the fall trial because the active correction (flexion) occurred later than in the recovery trial.

The difference between passive and active phases is illustrated in Figure 6 based on single trial responses to fast forward translations. It illustrates a typical successful trial (i.e., a recovery) compared to a fall after a fast-forward translation (backward fall). The first corrective step consisted of a passive extension followed by a fast flexion. Typically this flexion occurred faster in the trials with recovery, as compared to the fall trials. In contrast, note that the passive period (0–160 ms here) was similar for both types of trials. The limbs extend at the knee and ankle joint levels in both trials but more so and longer in falls. When all trials were considered, the ankle extended passively (plantar flexion) to a peak for 144 ms (SD 8 ms) in successful trials vs. 184 ms (SD 27ms) in fall trials. This difference was significant (*p* = 0.002) as was the difference in maximum added extension angle [4.5 (SD 1.3) and 8 (SD 1) degrees respectively; *p* = 0.003]. For the knee, a similar difference existed but it was not significant (peaks at 122 ms and 139 ms for recoveries and falls, respectively (*p* = 0.1), associated to peak extension magnitudes of 3 and 2° (*p* = 0.06). These data are indicative of different stiffness states at the onset of the perturbation (see Discussion).

A detailed analysis of the corrective responses and the discriminative variables between a fall and a recovery forms the subject of a subsequent paper but the data were in line with this typical result, shown here.

For forward fast trials, the head displacement was stabilized in space for a longer interval of time in recovery trials (298 ms, SD 20 ms) than in fall trials (205 ms, SD 14 ms).

Regarding the upper limbs, the data were very different than for the legs, as the expected passive part of their movement (pendulum-like) was barely visible to be analyzed. Overall, we observed early startle-like muscular activities, or functional movements (either flexion or abduction) for both outcomes (falls and recoveries).

During fast backward translations, only a few falls were observed (see earlier). The body swayed forward, leading to an anterior position of the CoM relatively to the base of support (BoS). A corrective step was then preferentially used to recover, and its absence was the signature of falls. Moreover, a startle-like arm movement was observed in fallers, with a significantly shorter latency of activation [F: 72 ms (SD 9) vs. Recovery (NC): 109 ms (SD 11), *p* = 0.02] but a smaller magnitude of activity (until 360 ms).

During slow anteroposterior translations (backward and forward trials), the CoM moved in phase with the platform and never crossed the BoS borders in recoveries. The head, trunk and limbs were kept aligned before a step occurred. Conversely, in falls, after slow translations, the body trajectory was en bloc and behaved as a non-controlled inverted pendulum.

## Discussion

The main findings of the present study were as follows. (1) By looking in detail to the movements of the body segments and their dispersion it appears that large corrective responses are observed only after 200 to 300 ms following the onset of perturbations, suggesting that inertia is the important element in this period (as confirmed with a 17-rigid-segment model) and that efforts aimed at recovery of falls should concentrate on the period 300–700 ms. (2) The head is the last segment to move thereby excluding an important role for vestibular and visual inputs for initiating the corrective responses. It leaves the proprioceptive sensors as sole source of information for 300 ms at the onset of unexpected fall. (3) Comparing the various directions of translations, the percentage falls for fast forward translations was highest [in line with (4)]. Better chances of recovery were seen when corrective steps were fast and infrequent. (4) With the randomization protocol used it took more than 20 trials to achieve a decrease in the rate of falls. These findings will be discussed in detail below.

### Actively preventing damage during a fall: a short time-window available

The pre-impact phase of a fall lasts some 700 ms in a standing subject (4). The present study shows that this period consists of a “passive phase” with an immobility period (T1) followed by a period of passive motion of the segments (“free fall”) (T2). The term “passive” refers to the absence of large corrective responses involving changes in head position, which can apply to active or inactive muscles (4). The first period (T1) reflects an inertial delay as the onset of the detectable movement of each segment of the body was in linear relation to their respective height. Then, once it moved, the segments trajectory was similar from one trial to another (T2), until the variability started to increase, reflecting gain of control (T3).

The first two phases (T1 and T2) were considered to be mostly passive, dictated primarily by the inherent inertia and tone of a poly-articulated body translated at its basis, as strongly suggested by the simulated response of a mechanical model. Properties such as stiffness and damping intrinsic to the joints and muscles appeared to play a major role at the beginning of a fall, as previously suggested for the regulation of quiet upright stance (22, 25, 38–43). In contrast, it is suggested that the third phase (T3) is concomitant to the moment when active adjustments can be made, a point that is of major importance in the context of applying martial arts (or other) techniques for safe falling (see introduction). The present study identified the onset of this third phase by applying a quantitative measure of dispersion.

The passive phase is limited to approximately 200–300 ms (with a fall lasting typically about 700 ms). The “loss of balance point” was always reached in fast trials, supporting the hypothesis that there is an incompressible time lag, as the body behaves like a mechanical model composed of interconnected viscoelastic masses. Such a passive phase has been observed or described by others as well (27, 30). In their modeling study, Bortolami et al. (38) showed that a period of 125 ms after perturbation onset is needed before forces are generated for the CoP to go past the CoM, with the body performing a forward falling motion. Altogether then, it appears that one can start applying motor strategies aimed at preventing a fall at around 300 ms after the onset of perturbation (with a fall lasting typically about 700 ms). This result agrees with a modeling study showing that it should take about 300 ms delay before a reaction to a lateral fall can intervene to decrease hip impact (44). This pluriarticulated biomechanical behavior may generate compensatory feedback for additional stabilization that leads to a more effective control of the whole CoM even if adjustments in timing response are limited (45).

Conversely, muscle activities are recorded early (from 60 ms) but the analysis of the body trajectory suggests that they are initially insufficient to counteract the biomechanical forces resulting from the imposed external destabilization. In other words, the muscle activities at play in the initial phase of the fall influence the stiffness and damping of joints as well as the postural tone, but do not reflect yet a functional and active recovery strategy. We argue that this passive phase could be reduced according to the initial conditions of rigidity for instance. A remaining question is whether it is deleterious for the subject to shorten this “refractory period” driven by mechanical properties (lowest energy cost, mutimodality updated afferences, extra time).

### What patterns of sensory information contribute to postural control during a fall?

In addition, the present data throw light on the sensory source of the corrective responses. In fact, the deformation of the pluri-articulated body is specific to the type of perturbation (46). Here, a platform translation sends a “shock wave” through the body in a caudo-cranial ascendant progression. In accordance with this observation, we suspect that the sensory inputs follows such a temporal sequence. In fact, the head is the last segment to be impacted by the perturbation as it is the farthest from the point of external force application. It implies that visual and vestibular signals are the last to be involved in the on-going fall. This conclusion is specific for the perturbations as used here but it is important since it was occasionally speculated that vestibular inputs could play a role (47).

The present study shows that vestibular input is an unlikely source for reactions to translations since the head remains stationary during the first 200 ms of a fall. This is in line with several studies showing that postural reactions persist in the absence of a normal vestibular system (see introduction). It takes an additional 6 ms for vestibular information to reach several cortical areas (48) and from there, 41 ms to produce external force at the ankle. Altogether, a minimum latency of 247 ms would be required for vestibular information to contribute to the recorded dynamic responses.

In studies with a vertical drop, the situation is different since the head movement could directly trigger muscle synergies in about 60 ms in a pathway over the vestibular nuclei (49–51), leading to a vestibular contribution at about 270 ms. These long latencies explain why vestibulospinal responses would not be instrumental to control the CoM at the onset of a postural perturbation (52–54). However, they would be crucial for head–stabilizing reactions (55) and the processing of the postural vertical information needed to realign the body after recovery (56).

A retinal slip signaling the onset of a fall also requires a head movement. Furthermore, the pathway involved would be slow unless a subcortical pathway is involved (12). The fastest pathway to trigger a postural reaction would then relay through the vestibular nuclei in 28 ms (57). According to one source, after adding 60 ms for a descending volley and 11 ms for the electro-mechanical coupling, a minimum of 300 ms would be required for visual afferences to modulate the postural response, using the accessory optic system (58). Supporting the late contribution of visual inputs, Marigold et al. (59) showed that saccades to the ground were not initiated before 350 ms, after the appearance of an unexpected obstacle.

In contrast, input from the feet starts very early on in the presently described type of perturbations. In particular, the foot started to move 6 ms after the onset of the support-surface translation, generating shear forces after the acceleration of the support-surface upon which the subjects were standing. They conveyed early information about load variations (60). Later, the flow of proprioceptive inputs gradually involved more proximal segments (e.g., pelvis, lumbar column, neck). A delay of 40 ms was required for afferent signals to reach the cortex (61) and 30 more milliseconds were needed to trigger EMG activities in lower-leg muscles (62). Since there was an additional 11 ms for electro-mechanical coupling (63), it would take at least 87 ms to generate forces at the ankle joint, based on proprioceptive information, which is close to the 90 ms latency of the first muscle activities observed here and in previous studies on recoverable falls (64–67). Even if the automated postural responses are mediated by the brainstem, they still have latencies of this type. It is worthwhile noting that such responses do not contribute immediately to “dispersion” and to an increase in variability in head trajectory in the period of 90–150 ms. Possibly this has to do with the stereotyped nature of these responses. Weerdesteyn et al. (68) showed that these responses were basically similar in trials where subjects were instructed to fall and trials with recovery (except for a change in amplitude).

More generally the present data indicate that the earliest muscle activations have a relatively small impact on head and body motions in the first 200–300 ms. Hence it is proposed that interventions on recovery of falls concentrate on the period 300–700 ms, a period which is still long enough to allow the application of safe landing techniques, such as used in martial arts [see introduction (7, 20, 68–73)].

Ultimately, we think that the first synergies aimed at restoring balance are triggered by the most reliable sensors, which detect the onset of the postural disturbance at the earliest possible latency. Our results support that somatosensory receptors encoded the perturbation characteristics and triggered the initial corrective responses (67) in accordance with several studies on balance in older subjects with and without diabetic peripheral neuropathy (74–77).

### Learning not to fall: the added-value of feed-forward mechanisms to an exclusive feedback-based postural control?

The purpose of the present work was to give a detailed picture at the stages before touchdown, to detect whether and when someone can intervene in changing the way he/she falls.

Two main postural mechanisms are used by the central nervous system (CNS) to maintain and restore balance during a perturbation: the anticipatory postural adjustments (APAs) and the compensatory postural adjustments (CPAs). Previously, it was assumed that small and/or predicted internal perturbations can be counteracted with a feed-forward control (APAs) whereas a feedback-based postural muscle activation (CPAs) is the main mechanism of balance restauration to cope with large and/or unexpected perturbations (78, 79). However, several studies reported an increase of efficiency in the reactive recovery response after unexpected perturbation training which challenged mechanisms for dynamic stability (70, 80).

As we were primarily interested in reactive behavior, care was taken to avoid feedforward mechanisms as much as possible using unpredictability and trials randomization to avoid anticipatory behavior. This was successful for the first twenty to twenty-five trials as there was no difference in the rate of falls, hence these trials will be discussed first. In these trials the recovery depended primarily on corrective responses and it is important to know how fast these reactions occur because one can hope to be able to change these reactions. In this sense, the present study is similar to the work by van Swigchem et al. (7), who showed that EMG amplitudes needed for a safe fall technique started as early as 180–190 ms after onset of the (sideways) fall. It was concluded that voluntary motor control is possible within the duration of a fall, even in inexperienced fallers. The present data (200 ms passive phase) are in line with this work and are of crucial significance for the debate whether humans can intervene in how they fall (Robinovitch, personal communication). It should be emphasized that the term “passive” is used here to indicate that there is no contribution of gross corrective responses. It does not exclude that there is a contribution of spinal stretch reflexes but these are limited to a contribution to local stiffness and have no effect on total body behavior. Nichols and Houk (81) showed that the spinal stretch reflex is well-suited to provide muscle stiffness at a time when inherent muscle stiffness fails. This reflex is an active process but it is a local phenomenon and is considered here as part of the passive phase, when whole body responses are considered.

After the first twenty trials there was evidence for “learning” since there was a drop of % falls and a decrease in the rate of falls variability. In this case, one can assume that there is an ability to acquire “fall-resisting skills” during repeated exposure to slips, which would also be generalizable (27). This phenomenon called habituation (82) indicates that subjects are not naïve any more to the upcoming perturbations and the familiarization with the disturbance is accompanied by a greater number of catch-ups: compensatory strategies employed are more effective at recovery, even when the perturbations were presented in random order or onset.

Weerdesteyn et al. (83) also observed a success rate of 17% in the first trial vs. 92% in their last trial. This attempt to reproduce a postural response that increase the likelihood of successful recovery may be related to a shift from a sensory feedback-control-related reactive response based on error correction in the preceding trials to an adaptative feedforward CNS control in order to proactively improve stability (27). The observation that randomizations of postural perturbation does not completely eliminate improvement in corrective responses is in line with previous work (19, 84).

The strategies used to prevent falls were not studied in details here but in the literature several options were suggested. Among the emerging postural adjustments that were described elsewhere on re-exposure to external perturbations, we can mention a pluri-articulated response at hip and ankle levels (85), a better regulation of the CoM position relative to the BoS (27, 45) and a decrease of the amplitude of postural reactions (82, 86). Also, a more flexed knee joint allows the COM to be lower, thereby increasing stability (87, 88). Alternatively, a stiffness strategy can be implemented through muscle co-contraction (agonists/antagonists) as was observed in challenging postural threat conditions (78, 89, 90), pre-programmed reactions (83, 91) or startle reactions (47, 92, 93). In older subjects, co-contraction about the ankle is often seen during static balance challenges but it was shown that this is not necessarily a predictor of successful fall avoidance in this population (94).

Further studies should focus on assessing if predictive adjustments are being made (pre-perturbation behavior such as a squatting initial posture, center of pressure displacements,.) supporting the fact that learning not to fall rely on this interplay between reactive and predictive adaptations (hybrid control theory). In addition to these experimental observations, some laboratory-based measures of postural control (posturography) would be of great interest to reveal subtle deficits in the underlying control mechanisms (95) as it is aknowledged that the inability to produce APA is related to an increased likelihood of falls if older adults (96) or multiple sclerosis patients (97). Finally, a change in central set can also influence the postural response and the outcome (98).

### Functional implications

The present data show that there is limited time to adjust the way one falls from a standing position. We showed that the pre impact period is truncated of 200 ms or so. During the initial part of a fall, the observed trajectory results from the interaction between the destabilizing external force and the body: the inertial properties intrinsic to joints, ligaments and musculotendinous system have then a major contribution, as suggested for the regulation of static upright stance. This passive phase is later followed by an active phase, which consists of a corrective response to the postural perturbation. Thus, we believe that during a fall from standing height, it takes about 300 ms for postural responses to start correcting the body trajectory, while the impact is expected (to occur) around 700 ms. It has been argued that this time is sufficient to change the way one falls and that this makes it possible to apply safer ways of falling (7, 69, 70), for example by using martial arts fall techniques (68, 71–73). Despite these constraints, our study also supports the idea that learning is possible even though it may take a large number of trials.

Currently the training with balance perturbations is increasingly popular. One example is the work of Dijkstra et al. (84). It is of interest to note that this study failed to show generalization of improvements in stepping responses (anterior-posterior perturbation training did not generalize for lateral translations) and the authors suggested that multidirectional training possibly could facilitate generalization. This is exactly what the current paper showed, as indeed improvement occurred for all types of perturbations despite randomization. However, it did take more than 25 trials to obtain this result. This is of great importance as it can indeed encourage people in this field to invest in multidirectional training protocols, provided they are willing to use extended training periods.

## Ethics statement

Human Ethics Committee on Human Research of the University of Paris 6 (CPP 06036).

## Author contributions

ML: principal work, redaction of the paper. DW: experiment, signal processing and helped with the redaction of the paper. EC: helped with signal processing of the EMG. JL: helped with EMG. SL: co-director of the PhD thesis for this work. JD: scientific expert, redaction of the paper. P-PV: director of this project, redaction of this paper. CV: help to do analysis of signal.

### Conflict of interest statement

The authors declare that the research was conducted in the absence of any commercial or financial relationships that could be construed as a potential conflict of interest.

## Abbreviations

ANOVA: analysis of variance
BoS: base of support
BMI: body mass index
CoM: center of mass
CoP: center of pressure
EMG: electromyography
DLT: direct linear transform
FS: fall score
F: fall
NF: recovery
PO: perturbation onset
SAS: statistical analysis software
SD: standard deviations
TTC: time to contact.
