# Corrigendum: An Initial Passive Phase That Limits the Time to Recover and Emphasizes the Role of Proprioceptive Information

**DOI:** 10.3389/fneur.2019.00118

**Published:** 2019-02-27

**Authors:** Maeva Le Goic, Danping Wang, Catherine Vidal, Elodie Chiarovano, Jennyfer Lecompte, Sebastien Laporte, Jacques Duysens, Pierre-Paul Vidal

**Affiliations:** ^1^COGNAC-G (COGNition and ACtion Group), Université Paris Descartes–CNRS UMR-MD–SSA, Paris, France; ^2^Institute of Information and Control, Hangzhou Dianzi University, Hangzhou, China; ^3^Plateforme d'Etude de la Sensorimotricité, Université Paris Descartes, Paris, France; ^4^Arts et Metiers ParisTech, Institut de Biomecanique Humaine Georges Charpak, Paris, France; ^5^Movement Control and Neuroplasticity Research Group, Department of Kinesiology, KU Leuven, Leuven, Belgium

**Keywords:** accidental fall, disequilibrium, stability, postural control, perturbation, sensory information, biomechanics, multi-joint kinematical model

“Catherine Vidal” was not included as an author in the published article. The corrected **Author Contributions Statement** appears below.

ML: principal work, redaction of the paper. DW: experiment, signal processing and helped with the redaction of the paper. EC: helped with signal processing of the EMG. JL: helped with EMG. SL: co-director of the PhD thesis for this work. JD: scientific expert, redaction of the paper. P-PV: director of this project, redaction of this paper. CV: help to do analysis of signal.

Additionally, in the published article, there was an error regarding the affiliations for Pierre-Paul Vidal. Instead of affiliations “1 and 2” it should be affiliations “2 and 1”.

The authors apologize for these errors and state that they do not change the scientific conclusions of the article in any way. The original article has been updated.

